# A Negative Relationship between Foliar Carbon Isotope Composition and Mass-Based Nitrogen Concentration on the Eastern Slope of Mount Gongga, China

**DOI:** 10.1371/journal.pone.0166958

**Published:** 2016-11-21

**Authors:** Jiazhu Li, Guoan Wang, Runan Zhang, Li Li

**Affiliations:** 1 Institute of Desertification Studies, Chinese Academy of Forestry, Beijing, 100091, China; 2 Collegeof Resources and Environmental Sciences, China Agricultural University, Beijing, 100193, China; Wageningen University, NETHERLANDS

## Abstract

Plants adopt ecological strategy to resist environmental changes and increase their resource-use efficiency. The ecological strategy includes changes in physiological traits and leaf morphology, which may result in simultaneous variations in foliar N concentration and the ratio of intercellular CO_2_ concentration to ambient CO_2_ concentration (c_i_/c_a_). This in turn links to foliar carbon isotope discrimination, and thus, a relationship between foliar N concentration and foliar carbon isotope composition (δ^13^C) is expected. To understand how plants integrate their structural and physiological resistance to environmental changes, the relationship between foliar N concentration and foliarδ^13^C has been assessed intensively, especially the correlation between area-based N concentration (N_area_) and δ^13^C.Less effort has been dedicated to the examination of the relationship between mass-based N concentration(N_mass_) and δ^13^C. Studies on the N_mass_–δ^13^C relationship, especially those including a large amount of data and species, will enhance our understanding of leaf economics and benefit ecological modeling. The present study includes an intensive investigation into this relationship by measuring foliar N_mass_ and δ^13^C in a large number of plant species grown on the eastern slope of Mount Gongga, China. This study shows that foliar N_mass_ decreases with increasing δ^13^C, which is independent of functional group, vegetation type, and altitude. This suggests that a negative correlation between N_mass_ and δ^13^C may be a general pattern for plants grown not only on Mount Gongga, but also in other areas.

## Introduction

Plants employ ecological strategy arising from modifications in physiological traits and leaf morphology to resist environmental changes. For example, variations in temperature lead to alterations infoliar Nconcentrations [1–3]. Since most N concentrates in the photosynthetic apparatus, photosynthetic ratesandthe ratio of intercellular CO_2_ concentration to ambient CO_2_ concentration (c_i_/c_a_) will change with varying foliar N concentrations. Foliar δ^13^C is related to photosynthetic rates and the c_i_/c_a_ ratio[4–6]; thus, δ^13^C changes with leaf N. Studying the relationship between N and δ^13^C could promote a better understanding of how plants adopt “ecological strategy” to resist environmental changes.

The uptake and assimilation of water, carbon, and nutrients by plants drives the biogeochemical cycles. Photosynthates and mineral nutrients, especially N, are invested in the construction of leaves, which in turn results in a new revenue stream of photosynthates to ensure continuity to future generations. All vascular plants engage in the same investment and re-investment processes [7]. Therefore, the inherent relationship between carbon, N, and water in plants is largely economic in nature. Leaf N and δ^13^C may serve as surrogates for N economy and water use efficiency (WUE), respectively. Thus, studies on the N–δ^13^C relationship in various species, functional types, and vegetation types in different biomes will contribute to a better understanding of resource utilization strategies adopted by plants.

Much research has been done on the relationship between N concentration and δ^13^C. Leaf N concentration can be expressed based on leaf area (N_area_) and leaf mass (N_mass_). In fact, the choice of mass-based or area-based expression of plant traits has been under discussion for a long time [8–13]. Although expressing leaf traits in terms of area seems intuitive and has always been adopted, mass-based expression has also been used intensively in recent decades [8, 9, 14–18] because it is easily quantified in terms of revenues and expenditures per unit investment [12]. Furthermore, mass-based expressions such as mass-based photosynthetic rate exhibit stronger correlations with foliar properties [10]. In addition, compared to area-based parameters, scientists have often preferred to implement mass-based parameters into ecological models because mass-based expression has shown a superior model fit [19]. Area- and mass-based measures can be interconverted via the leaf mass per unit area (LMA), and they both can be enlightening in complementary ways; hence, Wright et al. [12] and Westoby et al. [13] suggested exploring leaf traits from both perspectives.

With regard to the relationship between foliar N and δ^13^C, a considerable number of studies have focused on the assessment of the relationship between N_area_ and leaf δ^13^C. Although this relationship depends on the plant species, genotypes, and plant functional groups considered, most studies revealed that N_area_ is positively related to δ^13^C [20–24].This indicates that WUE increases with increasing N_area_. However, only a few studies have focused on the relationship between N_mass_ and δ^13^C, and these have been inconclusive. Two patterns have been reported—negative correlation [23, 24] and no correlation [25, 26].Thus, there is no conclusive statement regarding how WUE responds N_mass_ as yet. As above mentioned, N_mass_ is easily quantified in terms of revenues and expenditures per unit investment relative to N_area_, so, revealing the N_mass_–δ^13^C relationship will benefita better and more comprehensive understanding of water-carbon-nitrogen utilization patterns adopted by plants. Moreover, studies on the N_mass_–δ^13^C relationship may enhance the construction of biogeochemical and ecological models. For example, N_mass_ and δ^13^C have been implemented into the plant production (or yield) models developed by Aparicio et al.[27] and Luo et al. [28] based on the tight correlations between photosynthetic rate and δ^13^C and leaf N. The limited datasets and species involved in the previous studies on N_mass_–δ^13^C relationship might account for the observed inconsistent results,andnew data including more species are therefore necessary.

Therefore, the present study presents an intensive investigation on the relationship between N_mass_ and δ^13^C by collecting a large number of plant samples on the eastern slope of Mount Gongga, China, which is characterized by a wide range of environmental conditions and abundant plant species.

Mountains have long been considered ideal sites for studying plant physiological and morphological responses to environmental factors because of the marked changes in climate with increasing altitude. Altitudinal trends have been observed in leaf morphology. Thus, leaf thickness generally increases with altitude as temperature decreases [22, 23]. Therefore, the internal diffusion pathway of CO_2_ from stomata to chloroplasts in leaves is longer in plants grown at high elevations, which leads to a lower CO_2_ supply at the site of carboxylation, a lower c_i_/c_a_ratio, and thus a less negative δ^13^C. In contrast, a thick leaf (i.e., a larger LMA) is always associated with a low N_mass_ [11, 15, 23, 25]. Therefore, we hypothesized that foliar δ^13^C is negatively related to N_mass_.

## Materials and Methods

No specific permits were required for the described field studies because the location is not privately owned or protected. Our studies did not involve endangered or protected species; thus, no relevant permissions/ permits were required for the field studies.

### Study area

Mount Gongga is located on the southeastern side of the Qinghai-Tibet Plateau in Southwest China (101°30ʹ–102°10ʹE, 29°20ʹ–30°00ʹN). Remarkable differences in terrain and climate exist between its eastern and western slopes. Our study was conducted on the eastern slopes, which have an altitude that spans from 1100 m above sea level (a. s. l.)(Dadu River Valley) to 7600 m a.s. l., and a climate that is warm and dry at low elevations and cold and moist at high elevations. On the slopes, the temperature decreases, while precipitation increases, with increasing altitude, as inferred from the records of two meteorological observatories located on the slopes and the regional hydrology data obtained by Zhong et al. [29]. The mean annual temperature and the mean annual precipitation are 12.2°C and1050 mm, respectively, at an altitude of 1640 m (altitude of the meteorological observatory Moxi) and 4.0°C and 1938 mm, respectively, at 3000 m (altitude of the ecological observatory Hailougou).

The eastern slopes of Mount Gongga have an intact and continuous vertical vegetation spectrum. It consists of subtropical evergreen broad-leaved vegetation (1100–2200 m), temperate coniferous and broad-leaved mixed forests (2200–2800 m), frigid dark coniferous forests(2800–3600 m), alpine sub-frigid shrub and meadow vegetation(3600–4200 m), alpine frigid meadow vegetation (4200–4600 m), alpine frigid sparse grass and desert zone(4600–4800 m), and alpine ice-and-snow zone(>4900 m). The vertical distribution of the soil on the eastern slope of Mount Gongga is also very pronounced, in a continuous soil sequence from 1100 m to 4900 m. It comprises yellow-red soil (luvisols) (<1500 m), yellow-brown soil (luvisols) (1500–1800 m), brown soil (1800–2200 m) (luvisols), dark-brown soil (luvisols) (2200–2800 m), dark-brown forest soil (luvisols) (2800–3600 m), black mattic soil (cambisols) (3600–4200 m), mattic soil (luvisols) (4200–4600 m), and chilly desert soil (cryosols) (> 4600 m).

### Plant sampling

An elevation transect spanning from 1200 to 4500 m a. s. l. was set on the eastern slope of Mount Gongga. Plants were sampled along the altitudinal transect at intervals of approximately 100 m in August 2004. The sampling was restricted to unshaded sites far from human habitats. Almost all the species that could be found at each sampling altitude were collected and 5–7 specimens of each species of interest were identified at each site. The same number of the uppermost leaves was sampled from each herb and shrub, whereas eight leaves were collected from each tree individual, two leaves at each of the four cardinal directions relative to the position of full-irradiance located about 8–10 m above the ground. The leaves from each species at each elevation were pooled for further measurements, resulting in a total of 460 samples.

### Measurements of foliarnitrogen contentand δ^13^C

The foliarsamples were oven-dried at 65°C and ground to pass through 40 mesh. Each ground foliar sample was divided into two parts, one for N_mass_ measurement and the other for δ^13^C measurement. Foliar N_mass_ was measured using an elemental analyzer (Flash EA1112, CE Instruments, Wigan, UK), with a combustion temperature of 1020°C. Urea, obtained from the International Atomic Energy Agency (IAEA), was taken as the laboratory standard matter. The standard deviation for this N_mass_ measurement was 0.1%, obtained by measuring the standard matter 46 times and the same foliar sample 10 times.

Foliar δ^13^C was determined on a Delta^Plus^XP mass spectrometer (Thermo Electron GmbH, Bremen, Germany) coupled with an elemental analyzer in continuous flow mode. About 250 μg of ground foliarmaterial was included in a tin capsule, which was placed in the elemental analyzer, where the sample was combusted at atemperature of 1020°C.The CO_2_ gas derived from the combustion was carried by helium gas into the Delta^Plus^XP mass spectrometer, where δ^13^C was determined. Glucose, obtained from the IAEA, was used as the laboratory standard matter. The standard deviation of δ^13^C was estimated at less than 0.15‰ by measuring the standard matter 46 times and the same foliar sample 10 times. The carbon isotopic ratios were reported in the standard notation relative to the Vienna Pee Dee Belemnite standard.

Detailed N_mass_ and δ^13^C values of foliar samples are shown in S1 Dataset.

### Data analyses

Although C_4_ plants and mosses were also collected, the relationship between foliar N_mass_ and δ^13^C was addressed for only C_3_ seed plants and ferns in the present study. Foliar N_mass_ was log_10_-transformed before statistical analyses to improve data normality. Differences of δ^13^C among various functional groups were analyzed using one-way ANOVA. As both axes are equally prone to error, to avoid biases of the slope estimates, reduced major axis (RMA) regression was performed to detect the linear relationships between N_mass_ and δ^13^C. In order to reveal the potential influences of functional group, vegetation type, and altitude on the relationship between N_mass_ and δ^13^C, we performed bivariate correlation analyses between N_mass_ and δ^13^C for all plants pooled together and for seed plants. We also performed partial correlation analyses between N_mass_ and δ^13^C while controlling for functional group, vegetation type, and altitude. Finally, we compared the results derived from the bivariate correlation analyses with those from the partial correlation analyses.

## Results

**Fig 1:**
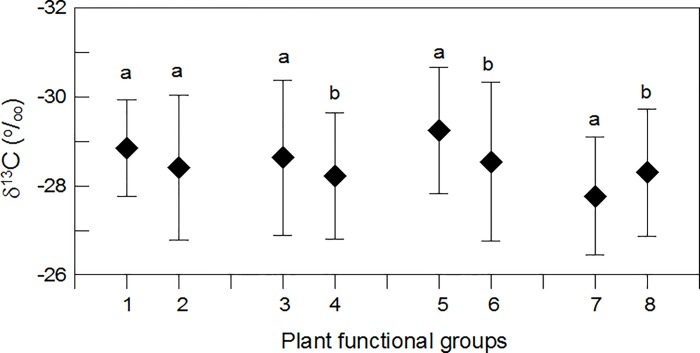
Mean leaf δ^13^C values ± 1SD across different plant functional groups. Different letters indicate significant difference at the 0.05 level. 1: ferns; 2: seed plants; 3: herbaceous plants; 4: woody plants; 5: annual herbaceous plants; 6: perennial herbaceous plants; 7: evergreen woody plants; 8: deciduous woody plants.

**Fig 2:**
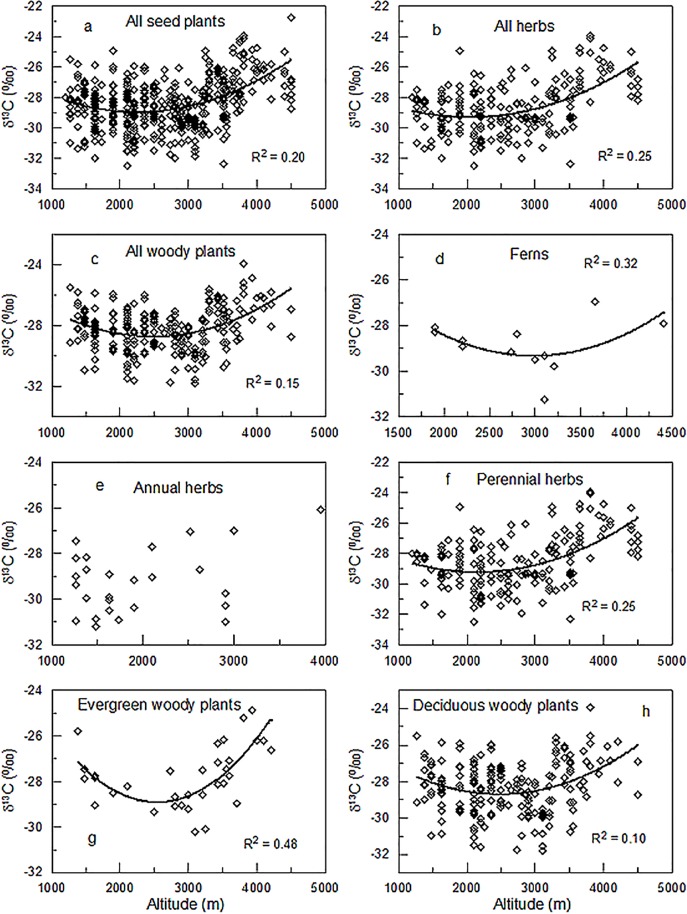
Variations in leaf δ^13^C with altitude for different functional groups. a: seed plants; b: herbaceous plants; c:woody plants; d: ferns; e:annual herbaceous plants; f:perennial herbaceous plants; g:evergreen woody plants; h: deciduous woody plants.

We performed a statistical comparison of δ^13^C among different functional groups using one-way ANOVA for the plants grown on the eastern slope of Mount Gongga. The statistical analysis revealed that functional group had a significant influence on leaf δ^13^C (*p*< 0.001) and that a significant difference existed between herbaceous plants and woody plants, annual herbaceous plants and perennial herbaceous plants, and evergreen woody plants and deciduous woody plants; no difference was observed between ferns and seed plants (Fig 1). However, functional group did not seem to exert an influence on the variation of δ^13^C with altitude, because all functional groups, except annual herbs, displayed a similar altitudinal pattern that leaf δ^13^C first decreased and then increased with increasing elevation (Fig 2).

**Fig 3:**
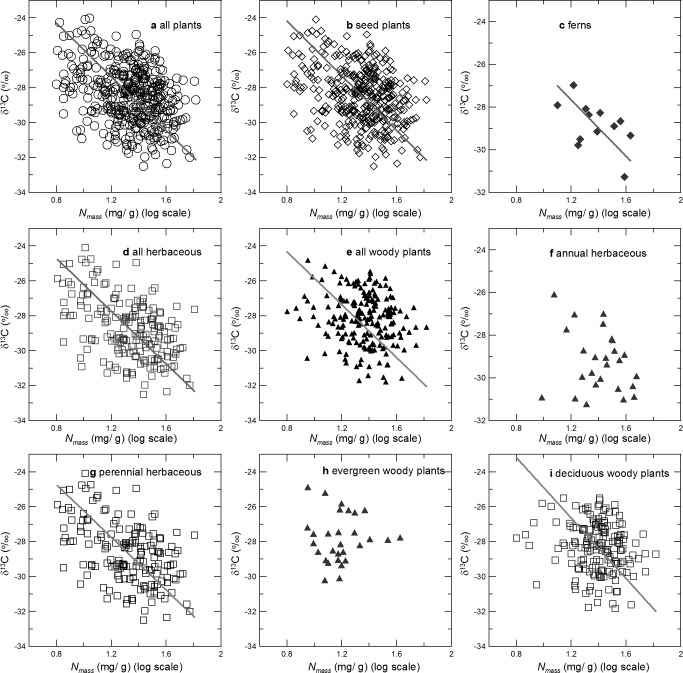
Correlation between leaf δ^13^C and leaf N_mass_ for different plant groups.

**Fig 4:**
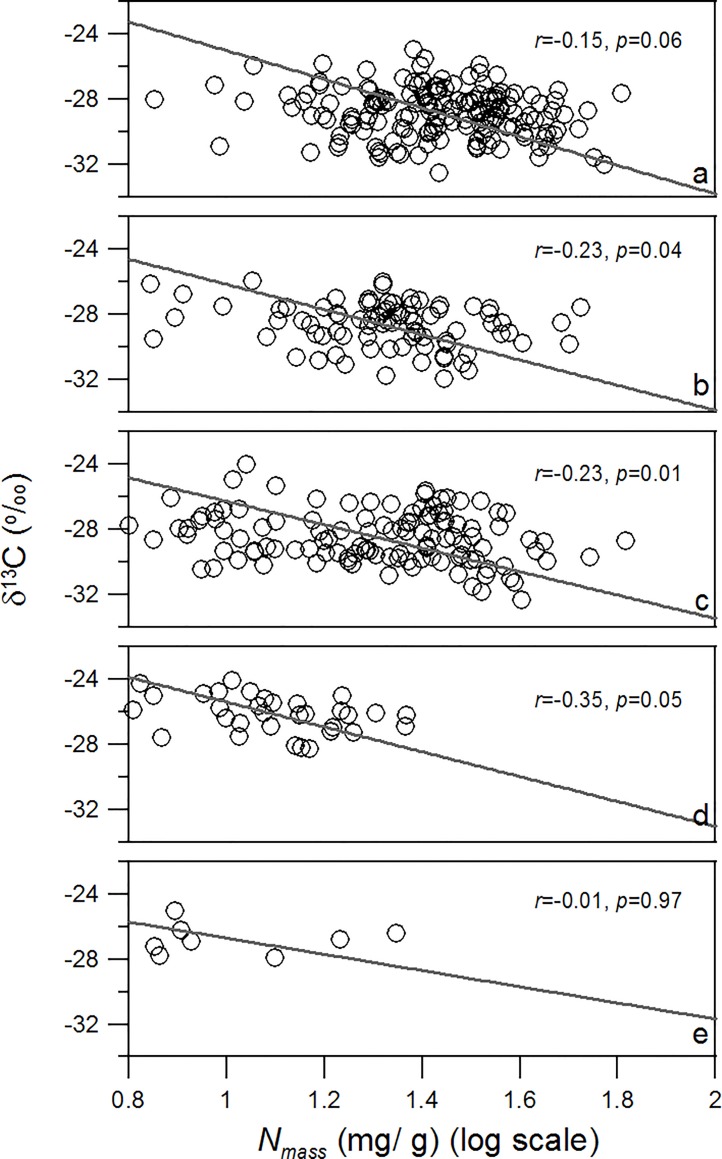
Correlation between leaf δ^13^C and leaf N_mass_ for different vegetation types. a: evergreen broad-leaved forests; b:coniferous and broad-leaved mixed forests; c:frigid dark coniferous forests; d:alpine sub-frigid shrub and meadow vegetation; and e:alpine frigid meadow vegetation. Solid lines indicate the linear regressions of δ^13^C vs. N_mass_.

An RMA regression of N_mass_ against δ^13^Cfor all plant samples pooled together revealed that δ^13^C significantly decreased as N_mass_ increased (*r* = -0.355, *p*< 0.001) (Table 1, Fig 3A).A series of RMA regressions were also conducted for each functional group and each vegetation type. A significant negative relationship between δ^13^C and N_mass_ was found for most of the functional groups studied, although the relationship was not significant for annual herbaceous (*r* = -0.234, *p* = 0.251) or evergreen woody plants (*r* = -0.003, *p* = 0.989), and it was only marginally significant for ferns (*r* = -0.521, *p* = 0.082)(Table 1, Fig 3B–3I). Negative relationships between δ^13^C and N_mass_ were also observed in most of the vegetation types studied, but some of the correlations were weak (e.g., *p* = 0.06 for evergreen broad-leaved forests and *p* = 0.97 for the alpine frigid meadow vegetation)(Table 2, Fig 4).

**Table 1:** Results of reduced major axis regressions of N_mass_ vs. δ^13^C for different plant groups.

Plants groups	intercept	Slope	*r*	*p*	N
All plants	-24.78	-2.74	-0.355	<0.001	426
Seed plants	-17.92	-7.83	-0.33	<0.001	414
Ferns	-19.76	-6.59	-0.52	0.082	12
Herbs	-18.67	-7.56	-0.47	<0.001	200
Woody plants	-17.34	-7.99	-0.18	<0.001	214
Annual herbs	-18.05	-7.95	-0.23	0.251	26
Perennial herbs	-18.70	-7.54	-0.48	<0.001	174
Evergreen woody plants	-38.09	-8.88	-0.003	0.989	29
Deciduous woody plants	-16.25	-8.66	-0.16	0.035	185

**Table 2:** Results of reduced major axis regressions of N_mass_ vs.δ^13^C for different vegetation types.

Vegetation type	intercept	slope	*r*	*P*
Evergreen broad-leaved forests	-16.22	-8.80	-0.15	0.062
Coniferous and broad-leaved mixed forests	-18.43	-7.74	-0.23	0.036
Frigid dark coniferous forests	-19.09	-7.20	-0.23	0.014
Alpine sub-frigid shrub and meadow vegetation	-17.78	-7.63	-0.35	0.051
Alpine frigid meadow vegetation	-21.74	-4.97	-0.01	0.970

For all plants pooled together, the bivariate correlation analysis between N_mass_ and δ^13^C and the partial correlation analyses between N_mass_ and δ^13^C after controlling for functional group, vegetation type, and altitude revealed a significant negative relationship (Table 3). The correlation coefficients (*r*) for the bivariate correlation analysis (-0.355, *p*<0.001) and the partial correlation analyses (-0.353,*p*<0.000) between N_mass_ and δ^13^C after controlling for functional group were similar. However, the correlation coefficients for the partial correlation analysis after controlling for vegetation type (-0.237,*p*<0.001) and altitude (-0.259,*p*<0.001) differed from that for the bivariate correlation analysis between N_mass_ and δ^13^C (*r* = -0.355) (Table 3).

**Table 3:** Comparison of the results of bivariate correlation analysis and partial correlation analyses of N_mass_ vs.δ^13^C after controlling for functional group, vegetation type, and altitude.

Plant groups	n	Variable controlled for	*R*	*P*
All plants	414	None	-0.355	<0.001
		Functional group	-0.353	< 0.001
		Vegetation type	-0.237	< 0.001
		Altitude	-0.259	< 0.001
Seed plants	402	None	-0.334	< 0.001
		Functional group	-0.341	< 0.001
		Vegetation type	-0.210	< 0.001
		Altitude	-0.237	< 0.001

The statistical analyses of the seed plants yielded similar results as those for all plants (Table 3).The bivariate correlation analysis and the three partial correlation analyses also revealed a significantly negative relationship between N_mass_ and δ^13^C. Controlling for the functional group resulted in almost no change in the coefficient for the bivariate correlation analysis (*r* = -0.334)and the partial correlation analysis (*r* = -0.341); however, correlation coefficients after controlling for the vegetation type (*r* = -0.210) and altitude (*r* = -0.237) were different from that for the bivariate correlation analysis between N_mass_ and δ^13^C(*r* = -0.334) (Table 3).

## Discussion

A general negative correlation between foliarN_mass_andδ^13^C was observed for all plants pooled and for most functional groups and most vegetation types, although no correlation was found for annual herbaceous, evergreen woody plants or alpine frigid meadow vegetation. These findings supported our hypothesis. Leaf δ^13^C in all functional groups, except annual herbs, first decreased and then increased with increasing altitude (Fig 2), while the altitudinal trend in leaf N_mass_ in all functional groups, except evergreen woody plants, was the opposite to the pattern observed for leaf δ^13^C (see Fig 2 in Shi et al., 2012). A negative relationship between leaf N_mass_ and δ^13^C can be easily observed from these two figures.

According to the sample size statistics, annual herbaceous and evergreen woody plants both had a smaller sample size than did the other functional groups. Similarly, the sample size of the alpine frigid meadow vegetation was also small relative to that of the other four vegetation types. Therefore, limited data might be responsible for the observed absence of significant relationships

The bivariate correlation analyses and the partial correlation analyses suggested that the significant negative relationship between N_mass_ and δ^13^C was independent of the functional group, vegetation type, and altitude, although vegetation type and altitude had some influence on the relationship. Thus, the negative relationship between N_mass_ and δ^13^C may also be a general pattern for plants growing in other regions. However, because of the specific environmental conditions present on Mount Gongga (for example, the increase of precipitation with altitude), more data and research are needed to arrive at a stronger conclusion. If a region has a decreasing altitudinal trend for precipitation, no relationship is expected between leaf N and δ^13^C because of the different influences of water availability on leaf N and δ^13^C. Foliar δ^13^C was consistently reported to be negatively related to precipitation [30–32], whereas, no consistent conclusion has been reached about the relationship between water availability and leaf N. For example, Han et al. [1] observed a negative correlation between mean annual precipitation and foliar N, while Wu et al. [33] reported no relationship between them. However, to our knowledge, almost all mountainous terrains are characterized by increased water availability with increasing altitude, and thus, the observed pattern of negative correlation between foliar N and δ^13^C can be extrapolated.

These results are consistent with studies by Hultine and Marshall [24] and Cordell et al. [23], in which N_mass_ was negatively associated with δ^13^C. However, neither Kloeppel et al. [25] nor Duursma and Marshall [21]found a significant negative relationship between N_mass_ and δ^13^C.The reason that this negative relationship between N_mass_ and δ^13^C was not observed in these studies may be owingtothe limited number of species being involved in their studies or the local environment and geography.

Foliar N_area_ and N_mass_ both express foliar N concentrations; however, there is a difference in their interpretations. A negative relationship could occur between the two expressions of leaf N concentration due to high leaf thickness, since a thick leaf always contains more N and more secondary metabolites (such as lignin) per unit leaf area. These secondary metabolites may dilute the N content per unit leaf mass, leading to a high N_area_ but a low N_mass_ [25]. For example, Cordell et al. [23] reported that the leaf thickness and the leaf N_area_ in *Metrosiderospolymorpha* increased, while the leaf N_mass_ decreased, with increasing elevation. After arranging the data from Luo et al. [28], who conducted their study on Mount Gongga, a negative correlation between the two expressions of N concentration could be found. Thus, foliar N_area_ and foliar N_mass_ exhibit opposite relationships with foliar δ^13^C. Since a positive relationship between δ^13^C and N_area_ was observed in most previous studies, it is not surprising that the relationship between δ^13^C and N_mass_ is negative.

A higher N_mass_ is always associated with a lower LMA [11, 15, 23, 25], indicating that leaves with a higher N_mass_ have lower thicknesses. These thinner leaves havea shorter internal diffusion pathway from stomata to chloroplasts and thus a greater gas conductanceand consequently a greater CO_2_ supply at the site of carboxylation, i.e., a larger c_i_/c_a_ ratio, and therefore a greater foliar δ^13^Cvalue [22, 20, 34, 25]. Alternately, a thin leaf, even though it has a greater N_mass_, may have less N per unit area (i.e., a lower N_area_) than a thick leaf with a smaller N_mass_. A lower N_area_ always implies lower photosynthetic capacity (on an area basis) [12, 35]; therefore, less CO_2_ is assimilated at the site of carboxylation, which leads to an increase in c_i_/c_a_ ratio and increased δ^13^C. Therefore, coordinated structural and physiological adaptations account for the negative relationship between N_mass_ and δ^13^C.

Foliar N concentration has been suggested to vary markedly across functional groups. For example, Han et al. [1] conducted a synthesis of 753 species from China andobservedsignificant differences in leaf N between seed plants and ferns, evergreen trees and deciduous trees, and among herbs, shrubs, and trees. Our previous study also demonstratedthat deciduous woody plants had a higher leaf N than evergreen woody plants on the eastern slope of Mount Gongga[2]. In addition, asignificant influence of functional group on leaf δ^13^C has been reported in previous studies [29, 36–39] and was also observed in this study (Fig 1). Since both foliar N and δ^13^C are associated with functional groups, this might result in varyingpatterns in the relationship between N and δ^13^C. However, the presence of anegative relationship between N and δ^13^C was indicated for all functional groups. Furthermore, the bivariate correlation analysis and the partial correlation analysis after controlling for functional group yielded similar results. Thus, this study strongly suggests that functional group did not play a role in the relationship between N_mass_ and δ^13^C. The cause for all functional groups showing a similar pattern for the relationship between N_mass_ and δ^13^C is that almost all functional groups displayed a similar altitudinal pattern for foliar N (see Fig 2 in Shi et al., 2012) and foliar δ^13^C (Fig 2).

Communities in which the life forms of the dominant plants are similar are generally treated as being of the same vegetation type. The influence of vegetation type on the relationship between foliar N and δ^13^C mainly reflects the effects of functional groups on this relationship. Since the pattern of the negative relationship between N and δ^13^C is independent of functional group,it is also not controlled by the vegetation type. Although the bivariate correlation analysis and the partial correlation analysis, after controlling for vegetation type, both resulted in a significantly negative relationship between N_mass_ and δ^13^C, the coefficient derived from the partial correlation analysis somewhat differed from that from the bivariate correlation analysis, suggesting that vegetation type does have some influence on the relationship between N_mass_ and δ^13^C. The cause for this is that a vegetation type often consists of more than one plant functional group, and different functional groups present different *r* values for the relationship between N_mass_ and δ^13^C (Table 3).

As stated above, there is a continuous vertical vegetation spectrum on the eastern slope of Mount Gongga; thus, the effect of altitude on the relationship between N_mass_ and δ^13^C is mainly due to the effect of vegetation types on this relationship. Since all vegetation types yield the pattern of a negative relationship between N and δ^13^C, this pattern is independent of altitude. However, the coefficients derived from the partial correlation analysis after controlling for altitude slightly deviated from that from the bivariate correlation analysis, this suggests that altitude would also have some influence on the relationship between N and δ^13^C. The reason for this is that vegetation types vary with altitudes, and the *r* value for the relationship between leaf N_mass_ and δ^13^C is vegetation-type specific(Table 3).

## Conclusions

We collected a large number of plant samples on the eastern slope of Mount Gongga, measured their foliar N_mass_ and δ^13^C values, and conducted a full assessment of the relationship between foliar N_mass_ and δ^13^C in the samples. This study shows that foliar N_mass_ decreases with increasing foliar δ^13^C, which is independent of functional group, vegetation type, and altitude. This suggests that the negative correlation between N_mass_ and δ^13^C may be a general pattern.

## Supporting Information

S1 Dataset N_mass_ and δ^13^C values of folia samples. (XLSX)
